# Production of bioactive cytokines using plant expression system for cardiovascular cell differentiation from human pluripotent stem cells

**DOI:** 10.1186/s13287-025-04424-0

**Published:** 2025-06-25

**Authors:** Kozue Murata, Kanae Takamura, Risa Watanabe, Akitomo Nagashima, Miho Miyauchi, Yoshiteru Miyauchi, Hidetoshi Masumoto

**Affiliations:** 1 Clinical Translational Research Program, Center for Biosystems Dynamics Research, RIKEN, 2-2-3 Minatojimaminami-Cho, Chuo-Ku, Kobe, 650-0047 Japan; 2 Department of Cardiovascular Surgery, Kyoto University Hospital, Kyoto, Japan; 3 Medical Core Project Dept, Mitsubishi Chemical Corporation, Yokohama, Japan

**Keywords:** Vascular endothelial growth factor (VEGF), Activin A, Cardiomyocyte, Endothelial cell, Induced pluripotent stem cell (iPS cell) (iPSC)

## Abstract

Bioactive cytokines such as vascular endothelial growth factor (VEGF) and Activin A are critical for the differentiation of stem cells into vascular endothelial cells and cardiomyocytes. However, production of the cytokines using conventional *Escherichia coli* or mammalian cell expression systems carries risks of immunogenicity and viral contamination. In this study, we developed a VEGF and Activin A plant expression system and demonstrated that plant-expressed VEGF and Activin A are as active as their commercial counterparts. We also showed that plant-expressed VEGF and Activin A are as efficient as human recombinant counterparts in inducing endothelial cells and cardiomyocytes from human pluripotent stem cells. These results suggest that plant-expressed VEGF and Activin A are promising alternatives for the safe and efficient production of cardiac cells, specifically cardiomyocytes and endothelial cells, for stem cell-based regenerative medicine.

**Supplementary Information:**

The online version contains supplementary material available at 10.1186/s13287-025-04424-0.

## Introduction

Several growth factors and cytokines such as vascular endothelial growth factor (VEGF) or Activin A have been shown to play critical roles in regulating stem cell differentiation [1]. In the cardiovascular field, VEGF is an essential factor for differentiation of stem cells into vascular endothelial cells and cardiomyocytes, and is used in various differentiation induction protocols [2–7]. Traditionally, VEGF has been produced using *Escherichia coli* or mammalian cell expression systems, but there are concerns about immunogenicity and viral contamination with these methods, limiting clinical applications [8]. Given the expanding role of human stem cell-derived cardiomyocytes and other cardiovascular cell types as therapeutic products for cardiac regenerative medicine [9], developing a safer and more scalable production method for clinical-grade VEGF is a pressing need. Activin is also a crucial mediator in the initial stages of cardiomyogenic differentiation from pluripotent stem cells to cardiac progenitor cells, and it has been reported to work in coordination with other growth factors such as Bone Morphogenetic Protein 4 (BMP4) and Fibroblast Growth Factors (FGF) to optimize cardiac differentiation [6, 10]. In clinical utilization, similar to VEGF, ensuring productivity and safety in its production process is a significant concern.

Plant expression systems possess several advantages over mammalian cell culture platforms, including lower production costs, scalability, and a significantly reduced risk of pathogenic contamination [11]. Expression of VEGF has been achieved in plant hosts such as *Nicotiana benthamiana* and barley, and expression of Activin A has been accomplished in *N. benthamiana*. However, there have been no previous reports verifying the ability of plant-derived cytokines to directly differentiate stem cells into clinically relevant lineages. In this study, we expressed recombinant VEGF and Activin A using *Agrobacterium* infiltration-based transient expression in *N. benthamiana* and evaluated their bioactivities as well as their potential to induce differentiation of human induced pluripotent stem cells (iPSCs) into cardiomyocytes and endothelial cells. This represents the first study investigating plant-based expression systems as an alternative platform for producing clinically relevant VEGF and Activin A for cardiovascular cell differentiation from human pluripotent stem cells for cardiac regenerative medicine. Our findings suggest that plant systems may provide a viable, safe, and scalable approach to generate cardiogenic growth factors to direct human pluripotent stem cell fate for future stem cell therapies.

## Results

### Characterization of plant-expressed VEGF and Activin A

**Fig. 1:**
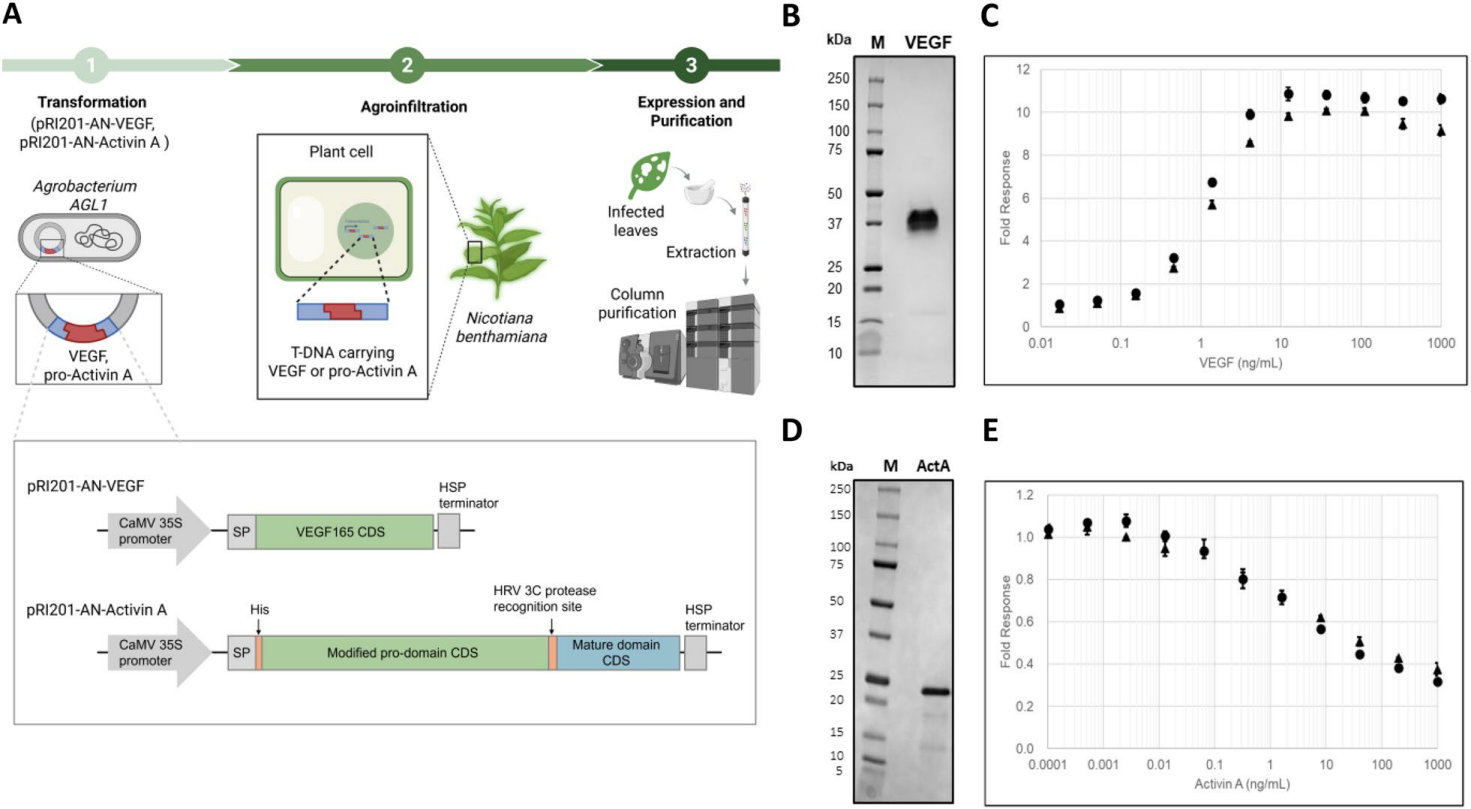
**A**: Schematic diagram of the plant protein expression system. **B**: SDS-PAGE analysis of plant-expressed VEGF (band around 37 kDa). A cropped gel image is shown. Full-length blots/gels are presented in Supplementary Fig. 1. Purified recombinant was separated under non-reducing conditions and stained with Coomassie Brilliant Blue G-250. **C**: Activity assay of plant-expressed VEGF. Data for the plant-expressed VEGF (closed triangles) and the standard VEGF used in the luciferase assay (Sf21-expressed, closed circles) are plotted as fold response over untreated (mean ± SD, n = 3). **D:** SDS-PAGE analysis of plant-expressed Activin A (band around 23 kDa). A cropped gel image is shown. Full-length blots/gels are presented in Supplementary Fig. 1. Purified recombinant was separated under non-reducing conditions and stained with Coomassie Brilliant Blue G-250. (M) Size marker, (ActA) Activin A. **E**: Activity assay of plant-expressed Activin A. MPC-11 cells were incubated with increasing concentrations of recombinant Activin A for 3 days before colorimetric measurement. Data for the plant-expressed Activin A (closed triangles) and standard Activin A used in this assay (CHO-expressed, closed circles) are plotted as fold response over untreated (mean ± SD, n = 4)

The plant expression vectors for VEGF and Activin A were constructed by inserting the nucleotide sequences encoding the native human VEGF165 protein with a rice α-amylase signal peptide at the N-terminus, or a modified pro-Activin A with a His-tag sequence and a few amino acid substitutions at the N-terminus, into the multiple cloning site of the pRI 201-AN vector (Takara Bio Inc., Kusatsu, Japan)(pRI 201-AN-VEGF/pRI 201-AN-Activin A). The modified pro-Activin A is processed during purification process by the HRV 3C protease to yield a mature Activin A variant with the second leucine residue substituted to proline. pRI 201-AN-VEGF and pRI 201-AN-Activin A were introduced into *Agrobacterium tumefaciens* AGL1 strain by electroporation, and co-infiltrated with a vector, pRIANP19, harboring the Tomato bushy stunt virus-derived gene silencing suppressor (TBSV P19) into *N. benthamiana* leaves using the agroinfiltration method. Six days after agroinfiltration, the infected leaves were harvested, frozen, ground, and extracted, followed by column purification (Fig. 1A). The recombinant VEGF165 dimer lacked the signal peptide at the purified stage, as confirmed by SDS-PAGE under non-reducing conditions and CBB staining (Fig. 1B). The activity of VEGF was measured using the VEGF Bioassay (Promega Corp., Madison, WI, USA) and found to be equivalent to that of Standard VEGF (Fig. 1C). The modified mature human Activin A exhibited the expected molecular mass on SDS-PAGE and CBB staining (Fig. 1D). The activity of the modified mature human Activin A was assessed by its inhibitory effect on MPC-11 cell proliferation and found to be comparable to that of Standard Activin A (Fig. 1E).

### Assessment of cardiomyocyte differentiation by plant-expressed VEGF

**Fig. 2:**
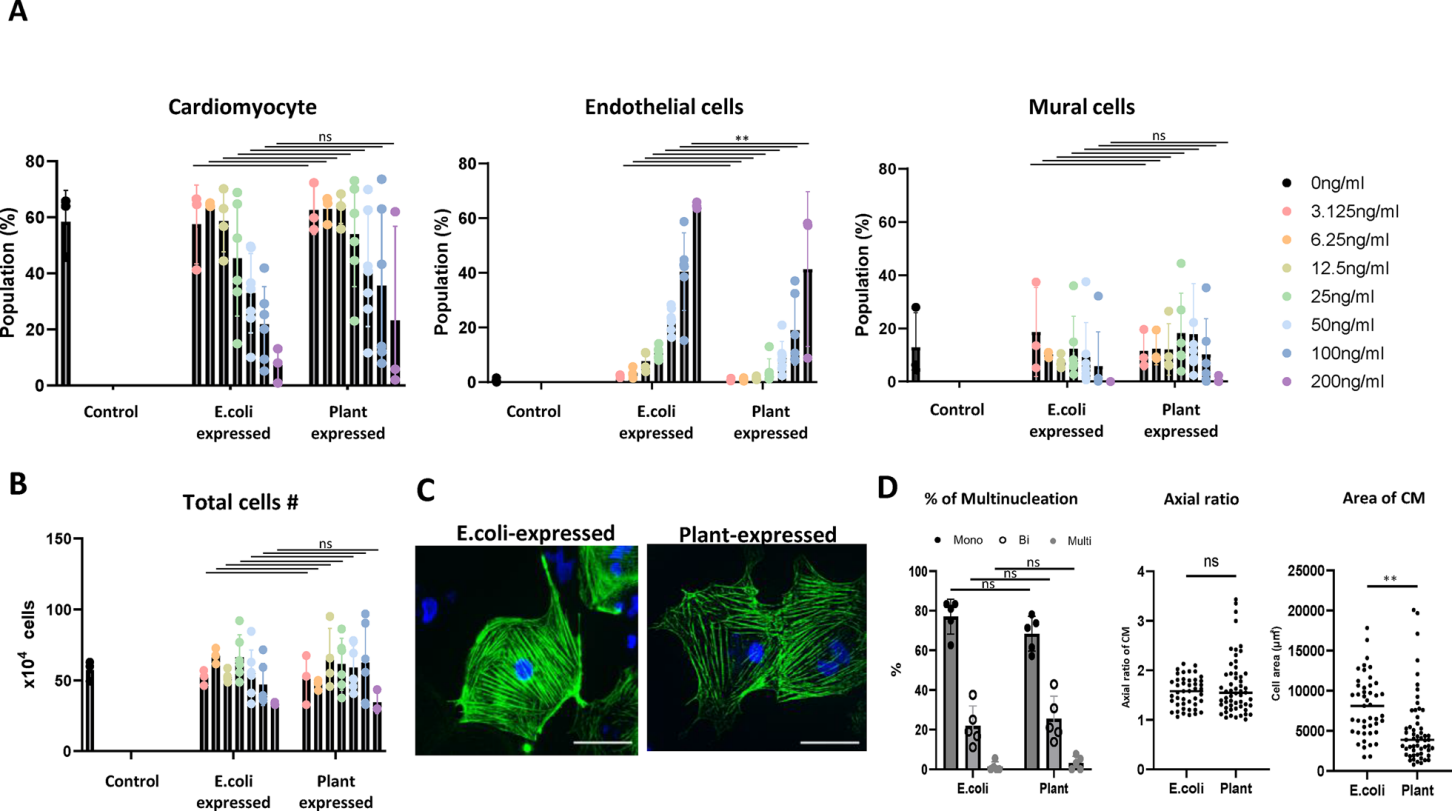
**A:** Differentiation efficiency of human iPSC-derived cardiomyocytes using plant-expressed VEGF (mean ± SD, n = 3–6), **: *P* < 0.01. **B**: Total cell number (mean ± SD, n = 3–6). **C**: Representative immunofluorescence images of cardiomyocytes. Green: cTnT, Blue: DAPI. Bar = 100 μm. **D**: Percentage of multinucleated cardiomyocytes (mean ± SD, n = 5), Axial ratio (n = 54–56), cell area (n = 54–56), **: *P* < 0.01

We investigated whether plant-expressed VEGF could induce cardiomyocyte differentiation from human iPSCs. Here, we employed the method we have previously reported, which induces differentiation of human iPSCs into both cardiomyocytes and vascular endothelial cells simultaneously [12, 13]. VEGF concentrations ranging from 0.625 to 200 ng/ml were used, with *E. coli*-expressed VEGF serving as a control. At lower concentrations, plant-expressed VEGF induced cardiomyocytes with similar efficiency as *E. coli*-expressed VEGF. For endothelial cell differentiation, plant-expressed VEGF was slightly less efficient compared to *E. coli*-expressed VEGF at the same concentration. (Fig. 2A). No significant difference in total cell number was observed in all concentration, indicating that the plant-expressed VEGF system does not reduce the viability of iPSC-derived cardiovascular cells (Fig. 2B). The differentiated cells expressed the cardiomyocyte marker cardiac troponin T (cTnT) and showed no significant differences in percentage of multi-nucleated cells, axial ratio compared to those induced with *E. coli*-expressed VEGF (Fig. 2C, 2D). However, the cardiomyocyte cell area analysis revealed that cells induced with *E. coli*-expressed VEGF exhibited a larger size compared to those induced with plant-produced VEGF (Fig. 2D).

### Assessment of endothelial cell differentiation induction by plant-expressed VEGF

**Fig. 3:**
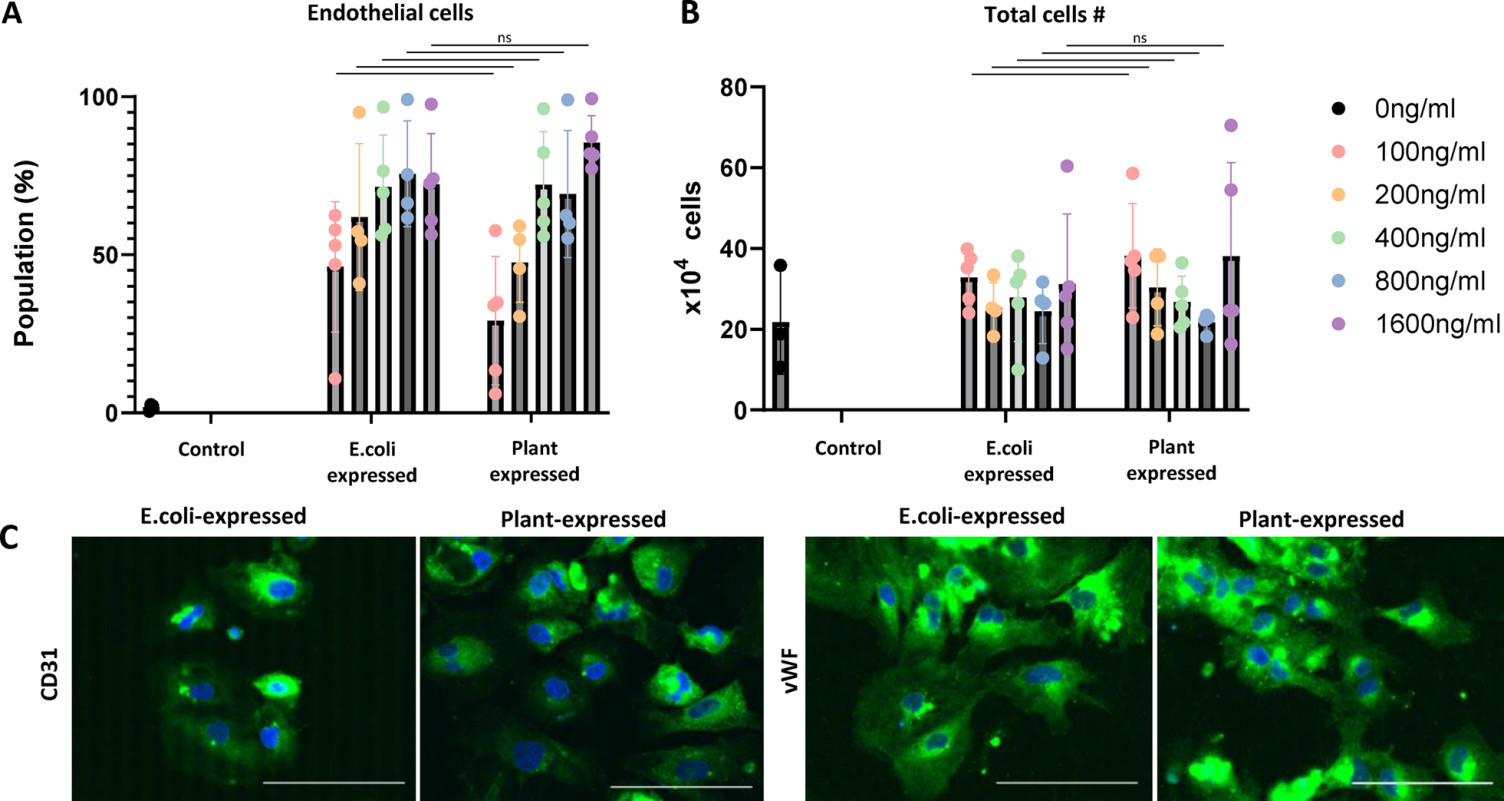
**A**: Differentiation efficiency of human iPSC-derived endothelial cells using plant-expressed VEGF (mean ± SD, n = 3–6). **B**: Total cell number (mean ± SD, n = 5). **C**: Representative immunofluorescence images of endothelial cells. Green: (left) CD31, (right) von Willebrand factor (vWF), Blue: DAPI. Bar = 100 μm

To evaluate the effects on non-cardiomyocyte lineages, we investigated whether plant-expressed VEGF could induce differentiation of human iPSCs into vascular endothelial cells using a previously reported defined endothelial differentiation protocol with modifications [14]. VEGF concentrations ranging from 100 to 1600 ng/ml were utilized, with *E. coli*-expressed VEGF serving as a control. At higher concentrations, plant-expressed VEGF induced endothelial cells with similar efficiency as *E. coli*-expressed VEGF (Fig. 3A). No significant difference in total cell number was observed in all concentration (Fig. 3B). The differentiated cells expressed canonical endothelial markers CD31 and von Willebrand factor (VWF), with no significant differences observed between conditions using *E. coli-* or plant-derived VEGF (Fig. 3C).

### Assessment of cardiomyocyte differentiation induction by plant-expressed Activin A

**Fig. 4:**
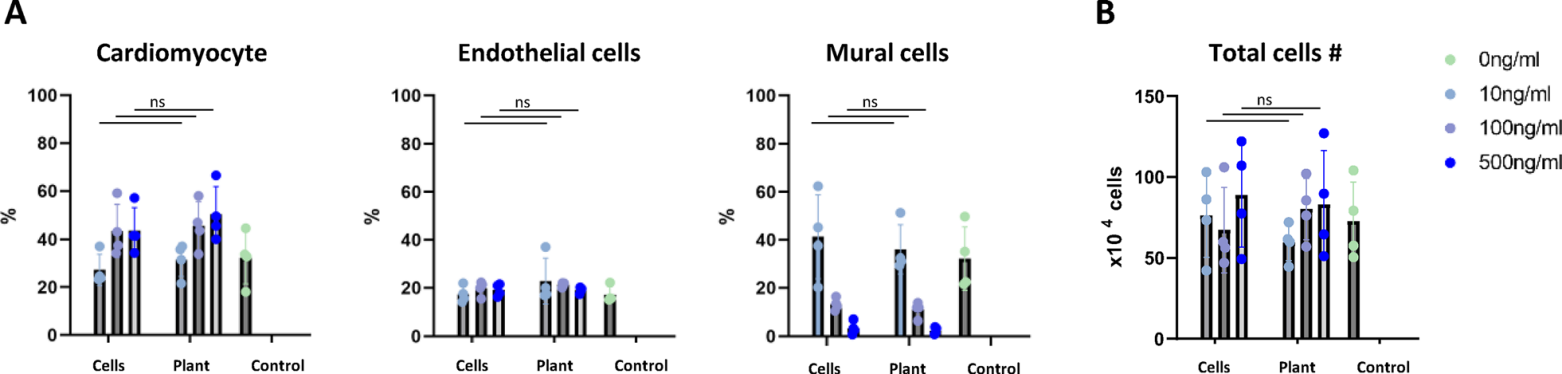
**A**: Differentiation efficiency of human iPSC-derived cardiomyocytes using plant-expressed Activin A (mean ± SD, n = 4). **B**: Total cell number (mean ± SD, n = 4)

In addition to VEGF, Activin A is also a critical factor for inducing differentiation of stem cells into cardiovascular lineages. We investigated whether plant-expressed Activin A could induce cardiomyocyte differentiation from human iPSCs [12, 13] Using Chinese hamster ovary (CHO) cell-expressed Activin A as a control, Activin A concentrations ranging from 10 to 500 ng/ml were tested. At higher concentrations, plant-expressed Activin A induced cardiomyocytes with similar efficiency as mammalian cell-expressed Activin A (Fig. 4A). No significant difference in total cell number was observed in all concentration, indicating that the plant-expressed Activin A system does not reduce the viability of iPSC-derived endothelial cells (Fig. 4B).

## Discussion

In this study, we have demonstrated that plant-expressed VEGF and Activin A exhibit biological activity equivalent to their *E. coli*-and mammalian cell-expressed counterparts in receptor binding assays. Furthermore, when human iPSCs were differentiated into cardiomyocytes and endothelial cells using plant-derived VEGF and Activin A, the differentiation efficiencies were on par with those achieved with factors derived from *E. coli* or mammalian cells. Notably, this is the first study to establish that plant-derived cytokines can effectively direct stem cell fate toward cardiovascular lineages. Immunofluorescence staining of cardiomyocytes differentiated with plant-expressed VEGF showed comparable expression levels of cardiac Troponin T, a cardiomyocyte-specific tropomyosin-binding subunit of the troponin complex which regulates cardiac muscle contraction. We observed no significant differences in the proportion of multinucleated cardiomyocytes or their axial ratio, both of which are indicators of cardiomyocyte maturity. These findings suggest that both *E. coli* and plant-expressed VEGF promote cardiomyocyte differentiation with a similar degree of maturation. While cells treated with E. coli-expressed VEGF exhibited a significantly larger cellular area, the largest cardiomyocytes within each population were observed in the plant-expressed VEGF group. This finding warrants further investigation to elucidate the potential impact of expression system-derived VEGF on cardiomyocyte hypertrophy. Furthermore, our results establish that plant-expressed VEGF and Activin A are equally effective in directing endothelial cell differentiation, with no discernible differences in differentiation efficiencies or histological morphology compared to conventional methods. This reinforces the viability of plant-derived cytokines as functionally equivalent alternatives for cardiovascular cell differentiation. Plant-based expression systems offer several advantages over bacterial and mammalian cell culture platforms, including reduced risk of immunogenicity, elimination of viral contamination concerns, and avoidance of endotoxin-related complications [15]. However, plant-specific post-translational modifications, particularly glycosylation patterns distinct from those found in human cells, remain a potential limitation. While such modifications could theoretically impact protein bioactivity or stem cell differentiation efficiency, our analyses indicate that the glycosylation profiles of the plant-expressed VEGF and Activin A do not impair their biological function. Additionally, plant systems enable cost-effective, scalable production of recombinant cytokines, presenting a compelling alternative to conventional platforms [16]. With the demand for stem cell-derived cellular products in regenerative medicine and drug discovery projected to surge to $49 billion globally by 2028 (“Regenerative Medicine Market,” Web site) [17], our findings underscore the transformative potential of plant-based cytokine production. As an economically and logistically viable alternative for generating clinical-grade growth factors and cytokines, our plant expression system could play a pivotal role in shaping the future landscape of stem cell-based regenerative medicine.

## Experimental methods

Details of the Experimental methods are described in Additional file 1.

## Supplementary Information

Supplementary file.1 (DOCX 25 KB)

Supplementary file.2 (DOCX 173 KB)

## Data Availability

The datasets used and/or analysed during the current study are available from the corresponding author on reasonable request. All experimental data are included in the manuscript.

## Abbreviations

VEGF: Vascular endothelial growth factor
iPSCs: Induced pluripotent stem cells
BMP4: Bone morphogenetic protein 4
FGF: Fibroblast growth factors
SDS-PAGE: Sodium dodecyl sulfate–polyacrylamide gel electrophoresis
CBB: Coomassie brilliant blue
cTnT: Cardiac troponin T
CHO: Chinese hamster ovary
