# Supplementary material for: Production of bioactive cytokines using plant expression system for cardiovascular cell differentiation from human pluripotent stem cells
Source: Stem Cell Res Ther. 2025 Jun 25;16:303. doi: 10.1186/s13287-025-04424-0 (PMC12188682; doi:10.1186/s13287-025-04424-0)
Supplement: Supplementary file 1 — Supplementary file.1 (DOCX 25 KB) [file 13287_2025_4424_MOESM1_ESM.docx]

Additional file 1

**Experimental methods**

**Vector preparation**

The manufacturing and production of all plant-expressed cytokines were carried out by Mitsubishi Chemical Corporation (Yokohama, Japan).

The VEGF165 expression vector (pRI 201-AN-VEGF) was constructed by synthesizing the nucleotide sequence encoding native human VEGF165 with a rice α-amylase signal peptide added to the N-terminus and inserting it into the multiple cloning site MCS1 of the pRI 201-AN plant expression vector (Takara Bio Inc., Kusatsu, Japan) in the forward orientation for VEGF165. The P19 expression vector (pRIANP19) was constructed by ligating the nucleotide sequence encoding the RNA silencing suppressor P19 derived from Tomato bushy stunt virus (TBSV) into the NdeI-SalI sites of the pRI 201-AN vector. The Activin A expression vector (pRI 201-AN-Activin A) was a modified pro-Activin A sequence described in the patent (WO2020-100993) with an N-terminal His tag, an 18 amino acid (PQGSLDTGEEAEEVGLKG) deletion, and a modified pro-Activin A sequence described in the patent (JP2020-156412) with substitutions to allow cleavage by the HRV 3C protease in place of Furin, were constructed by forward insertion into MCS1 of pRI 201-AN.

**Agrobacterium transformation and transient expression of recombinant proteins in *Nicotiana benthamiana***

The constructed vectors were introduced into Agrobacterium tumefaciens AGL1 strain by electroporation, and co-infiltrated with AGL1 harboring pRIANP19 into *N. benthamiana* plants using the agroinfiltration method. At 28 days after sowing, the *N. benthamiana* plants were inverted and the leaves were submerged in a beaker containing the Agrobacterium suspension. The beaker was placed in a vacuum desiccator (FV-3P, Tokyo Rikakikai Co., Ltd., Tokyo, Japan) and vacuum was applied to -0.09 MPa for 1 minute. The vacuum was then rapidly released by opening the valve. After releasing the vacuum, the plants were returned to an upright position and transferred to a growth chamber (LH-410SP, Nippon Medical & Chemical Instruments Co., Ltd., Osaka, Japan) set to 20°C, 60-80% relative humidity, 500 ppm CO_2_, continuous illumination with an average photosynthetic photon flux density (PPFD) of 140-160 μmol m^-2^ sec^-1^ using tri-wavelength fluorescent lamps (Rupikaline, Mitsubishi Electric Corp., Tokyo, Japan).

**Protein extraction and purification methods**

VEGF - 6 days after agroinfiltration, the infiltrated *N. benthamiana* leaves were harvested and frozen at -80°C. The frozen leaves were ground with 2x volume of extraction buffer (100 mM citrate buffer, 500 mM arginine, 2 mM sodium pyrosulfite, pH 4.0) using a homogenizer (T-25 digital ULTRA-TURRAX, IKA, Staufen, Germany). The extract was centrifuged to remove the plant debris containing insoluble proteins. The supernatant was filtered through a 0.22 μm bottle-top filter to prepare the sterilized pre-purification solution. VEGF purification was performed by the method described in the patent (JP2023-124843). The pre-purification solution was loaded onto column packed with TOYOPEARL Sulfate-650F (Tosoh Corp., Tokyo, Japan) pre-equilibrated with the extraction buffer. The column was washed with extraction buffer, wash buffer A (20 mM citrate buffer, pH 4.0), wash buffer B (20 mM potassium phosphate buffer, pH 7.0) and wash buffer C (20 mM potassium phosphate buffer, 400 mM NaCl, pH 7.0, 30%[v/v] acetonitrile) consecutively, and then VEGF was eluted with elution buffer (20 mM phosphate buffer, 500 mM NaCl, pH 7.0, 30%[v/v] acetonitrile). The eluted VEGF was diluted 20 times with binding buffer (20 mM glycine buffer, 150 mM NaCl, pH 10.0), and further purified by loading onto a HiTrap Capto Adhere (Cytiva) pre-equilibrated with binding buffer. The column was washed with binding buffer, wash buffer D (20 mM glycine buffer, pH 10.0), and wash buffer E (20 mM potassium phosphate buffer, pH 7.5) consecutively, and then VEGF was eluted with a 0-1000 mM NaCl linear gradient (20 CV).

Activin A - Infiltrated *N. benthamiana* leaves were harvested 6 days after infiltration and stored at -80°C until extraction. The frozen leaves were ground using a homogenizer (T-25 digital ULTRA-TURRAX) while frozen. The extraction buffer was 0.1 M sodium phosphate, 0.5 M arginine, 5 mM sodium pyrosulfite, pH 8.0 at 2x leaf weight. Ammonium sulfate was added to the recovered supernatant to 35% saturation and stirred at room temperature for 1 hour before centrifuging at 12,000 g for 15 min at room temperature to recover the 35% ammonium sulfate supernatant fraction. Ammonium sulfate was added to 60% saturation, stirred for 1 hour at room temperature, and centrifuged at 12,000 g for 15 min at room temperature to recover the 60-90% ammonium sulfate precipitate fraction. The precipitate was dissolved in His-tag affinity purification equilibration buffer (20 mM HEPES, 150 mM NaCl, 10% (w/v) glycerol, pH 8.0) and loaded onto a HisTrap HP 1 mL column (Cytiva) pre-equilibrated with the same buffer. The column was washed with His-tag wash buffer (20 mM HEPES, 150 mM NaCl, 20 mM imidazole, 10% (w/v) glycerol, pH 8.0), and the modified pro-Activin A was eluted with His-tag elution buffer (20 mM HEPES, 150 mM NaCl, 200 mM imidazole, 10% (w/v) glycerol, pH 8.0). HRV 3C protease was added to the eluted pro-Activin A to cleave the pro-domain. The crude Activin A was further purified by loading onto a SP Sepharose HP column (Cytiva) pre-equilibrated with 20 mM glycine pH 3.0, 40% (v/v) acetonitrile, and eluting with a 0-500 mM NaCl linear gradient (50 CV). The eluted fractions were pooled to obtain the purified modified Activin A. The purified Activin A was diluted 2-fold with 20 mM glycine pH 3.0, 250 mM NaCl and buffer exchanged into 4 mM HCl using an ultrafiltration concentrator (Amicon Ultra-4 10K, Merck, Darmstadt, Germany).

**Bioactivity assessment assays**

The activity evaluation of all plant expression system cytokines was conducted by Mitsubishi Chemical Corporation.

VEGF - The activity of recombinant VEGF proteins was analyzed using the VEGF Bioassay (Promega Corp., Madison, WI, USA), according to manufacturer’s instruction. In brief, VEGF Bioassay Cells were incubated with recombinant VEGFs at various concentrations. After 6-hour incubation, whole cell lysates were applied for luminescence quantification by luminometer. All test preparations including the VEGF standard (R&D Systems Inc., Minneapolis, MN, USA) were assayed in triplicate.

Activin A - The activity of recombinant Activin A proteins was detected by the inhibitory effect to MPC-11 cell proliferation[1]. MPC-11 was maintained in Dulbecco’s modified Eagle’s medium (DMEM; Thermo Fisher Scientific, Waltham, MA, USA) supplemented with 20% heat-inactivated horse serum and 1% penicillin-streptomycin. For experiments, MPC-11 cell suspension was added to 96-well culture plates at a density of 2000 viable cells/well in culture medium containing recombinant proteins at several concentrations for 3 days, followed by colorimetric detection of cell viability through Cell Counting Kit-8 (Dojindo; Kumamoto, Japan). All test preparations including the Activin A standard (R&D) were assayed in quadruplicate.

**Maintenance of human iPS cells (iPSCs) and differentiation of cardiovascular cell lines**

The present study used human iPSCs line 201B6 established at the Center for iPS Cell Research and Application (CiRA, Kyoto, Japan). The maintenance of human iPSCs and differentiation of cardiovascular cells was conducted in accordance with our previous studies[2–4] with modifications. In brief, iPSCs were expanded and maintained with StemFit AK02N medium (AJINOMOTO, Tokyo, Japan). At confluence, the cells were dissociated with TrypLE Select (Thermo Fisher Scientific), dissolved in 0.5 mM ethylenediaminetetraacetic acid in PBS (1:1) and passaged as single cells (5,000 – 8,000 cells/cm2) every 7 days in AK02N containing iMatrix-511 silk (FUJIFILM Wako Pure Chemical Corp., Osaka, Japan) (0.125 µg/cm2) (uncoated laminin fragment5) and ROCK inhibitor (Y-27632, 10 µM, FUJIFILM Wako). For cardiovascular cell differentiation, single iPSCs were seeded onto Matrigel-coated plates (1:60 dilution) at a density of 300,000–400,000 cells/cm2 in AK02N with Y-27632 (10 µM). At confluence, the cells were covered with Matrigel (Corning, NY, USA) (1:60 dilution in AK02N) one day before induction. We replaced the AK02N medium with RPMI + B27 medium (RPMI 1640, Thermo Fisher; 2 mM L-glutamine, Thermo Fisher; 1× B27 supplement without insulin, Thermo Fisher) supplemented with 10-500 ng/mL Activin A (R&D or Mitsubishi Chemical Corporation, Tokyo, Japan) (differentiation day 0; d0) and 5 µM CHIR99021 (Tocris Bioscience, Bristol, UK) was added for 24h, which was followed with supplementation with 10 ng/mL bone morphogenetic protein 4 (BMP4; R&D) and 10 ng/mL basic fibroblast growth factor (bFGF; FUJIFILM Wako) (d1) for 4 days without culture medium change. At d5, the culture medium was replaced with RPMI1640 medium supplemented with 0.625-200 ng/ml of vascular endothelial cell growth factor (VEGF)165 (FUJIFILM Wako or Mitsubishi Chemical Corporation), 2.5 µM IWP4 (REPROCELL, Yokohama, Japan) and 5 µM XAV939 (Merck). The culture medium was refreshed with RPMI1640 supplemented with 0.625-200 ng/ml VEGF every other day. Beating cells appeared at d11 to d15. In this protocol, we could exclusively induce cardiomyocytes (CMs) and vascular endothelial cells (ECs). To evaluate the differentiation efficiency of ECs, a part of the differentiation culture was changed to a method specialized for EC differentiation: after d5, the culture medium was replaced with RPMI1640 medium supplemented with 100-1600 ng/ml of VEGF165, 1 mM 8-Bromoadenosine-3',5'-cyclic monophosphate sodium hydrate (cAMP) (Nacalai Tesque, Inc., Kyoto, Japan) and was refreshed every other day with RPMI1640 supplemented with 100-1600 ng/ml VEGF.

**Flow cytometry**

Flow cytometry was conducted in accordance with our previous study with modifications[2]. Differentiated cardiovascular cells and cardiac tissue sheets were dissociated by incubation with Accutase and stained with one or a combination of the following surface markers: anti-PDGFRβ conjugated with phycoerythrin (PE), clone 28d4, 1:100 (BD) for MCs, and anti-VE-cadherin conjugated with phycoerythrin (PE), clone 55-7h1, 1:100 (BD) for ECs. To eliminate dead cells, cells were stained with the LIVE/DEAD fixable Aqua dead cell staining kit (Thermo Fisher). For cell surface markers, staining was carried out in PBS with 5% FBS. For intracellular proteins, staining was carried out in cells fixed with 4% paraformaldehyde (PFA) in PBS. Cells were stained with the anti-cardiac isoform of troponin T (cTnT) (clone 13-11) (Thermo Fisher) labelled with APC using Zenon technology (Thermo Fisher) (1:50) for CMs. The staining was performed in PBS with 5% FBS and 0.75% saponin (Nacalai Tesque). The stained cells were analyzed by CytoFLEX S (Beckman Coulter, Brea, CA, USA). Data was collected from at least 10,000 events. Data was analyzed with CytExpert software (Beckman Coulter).

**Immunofluorescence analysis (IFA)**

For IFA, CMTs were stained with cTnT antibody (Thermo Fisher) (1:250), CD31 (monoclonal mouse IgG1, clone 9G11) (R&D) (1:250) with DAPI (4‘,6-diamidino-2-phenylindole) (Thermo Fisher) (1：1000). Anti-mouse Alexa 546 (Thermo Fisher), anti-rabbit Alexa 488 (Thermo Fisher) and anti-mouse Alexa Fluor 488 (Thermo Fisher) were used as secondary antibodies. The tissues were photographed with an all-in-one fluorescence microscopic system, BZ-X800E (Keyence, Osaka, Japan) Combined Z-stack and sectioning functions. All results were confirmed with >2 repetitive independent experiments.

**Statistical analysis**

All statistical analysis was performed using Prism 9 (GraphPad, Boston, MA, USA) by one-way ANOVA or Kruskal-Wallis test with two-tailed P values, assuming parametric data for samples ≥ 6 and nonparametric data for samples < 6. No blinded tests were used to procedure the samples.

**Additional file reference**

1. Phillips DJ, Brauman JN, Mason AJ, De Kretser DM, Hedger MP. A sensitive and specific in vitro bioassay for activin using a mouse plasmacytoma cell line, MPC-11. J Endocrinol. 1999;162:111–6.

2. Masumoto H, Ikuno T, Takeda M, Fukushima H, Marui A, Katayama S, et al. Human iPS cell-engineered cardiac tissue sheets with cardiomyocytes and vascular cells for cardiac regeneration. Sci Rep. 2014;4:1–7.

3. Masumoto H, Nakane T, Tinney JP, Yuan F, Ye F, Kowalski WJ, et al. The myocardial regenerative potential of three-dimensional engineered cardiac tissues composed of multiple human iPS cell-derived cardiovascular cell lineages. Sci Rep [Internet]. 2016;6:1–10. http://dx.doi.org/10.1038/srep29933

4. Kawatou M, Masumoto H, Fukushima H, Morinaga G, Sakata R, Ashihara T, et al. Modelling Torsade de Pointes arrhythmias in vitro in 3D human iPS cell-engineered heart tissue. Nat Commun. 2017;8:1–11. http://dx.doi.org/10.1038/s41467-017-01125-y
