# Supplementary material for: Production of bioactive cytokines using plant expression system for cardiovascular cell differentiation from human pluripotent stem cells
Source: Stem Cell Res Ther. 2025 Jun 25;16:303. doi: 10.1186/s13287-025-04424-0 (PMC12188682; doi:10.1186/s13287-025-04424-0)
Supplement: Supplementary file 2 — Supplementary file.2 (DOCX 173 KB) [file 13287_2025_4424_MOESM2_ESM.docx]

Additional file 2

**Supplementary Figures**


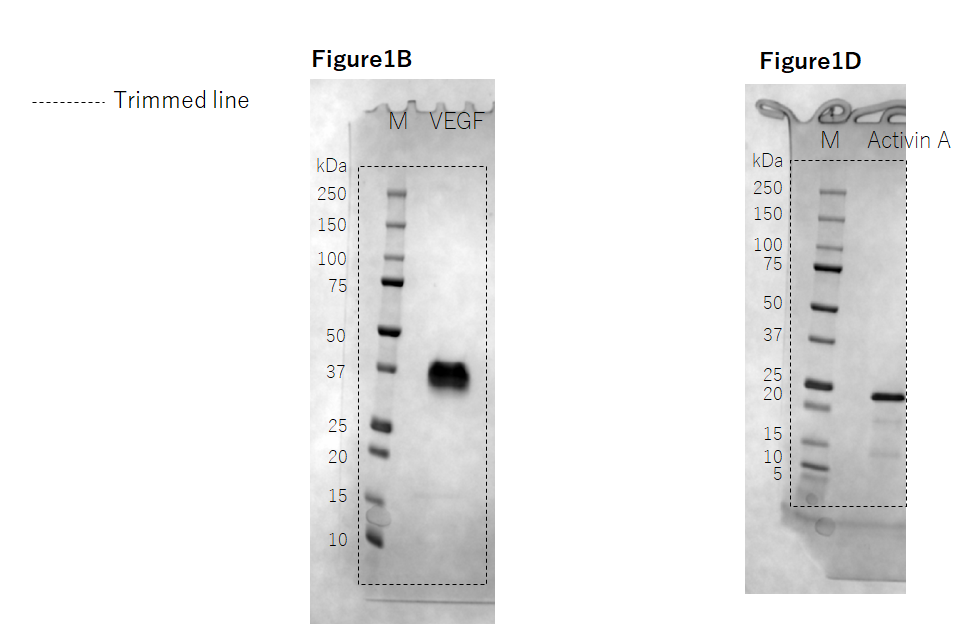


**Supplementary Figure 1: Full-length blots/gels for Figure 1B and D.**
